# Shotgun Metagenomic Characterization of Skin Microbiome Shifts in Human Scabies Before and After Scabicidal Treatment

**DOI:** 10.3390/ijms27188397

**Published:** 2026-09-21

**Authors:** Mingyu Kim, Woo Hyun Song, Hyun Jeong Ju, You Kyung Koh, Seong Joo Kim, Young Bok Lee, Minho Lee

**Affiliations:** 1 Department of Dermatology, Uijeongbu St. Mary’s Hospital, The Catholic University of Korea, Seoul 11765, Republic of Korea; kmg12058@gmail.com; 2 Department of Life Science, Dongguk University-Seoul, Goyang 10326, Republic of Korea; 3 Department of Dermatology, St. Vincent’s Hospital, College of Medicine, The Catholic University of Korea, Seoul 16247, Republic of Korea

**Keywords:** dysbiosis, metagenomics, scabies, skin microbiome, staphylococcus

## Abstract

Scabies, caused by *Sarcoptes scabiei*, is a globally prevalent ectoparasitic infestation associated with intense pruritus and secondary bacterial infection, yet the molecular composition of the skin microbiome during active infestation remains poorly characterized. We performed shotgun metagenomic sequencing of 41 skin samples collected from 18 patients at dry and moist anatomical sites before and after scabicidal treatment. In exploratory group-level comparisons, pretreatment moist-site samples had lower alpha diversity and higher bacterial and viral read-based burdens than post-treatment moist-site samples. Pretreatment dry and moist samples did not differ significantly in diversity, and *Staphylococcus* was the predominant genus. No genus- or species-level taxon or predicted pathway remained statistically significant after Benjamini–Hochberg false discovery rate correction at a threshold of 0.05. Nominal differences in predicted purine biosynthesis pathways were interpreted as exploratory observations. These findings provide a shotgun metagenomic characterization of the skin microbiome during active scabies and describe exploratory treatment-associated patterns that require confirmation in larger, paired longitudinal studies.

## 1. Introduction

Scabies is an infestation by *Sarcoptes scabiei var. hominis*, a burrowing mite that causes intense nocturnal pruritus and is highly contagious, making it a growing global health concern [1,2]. Clinically, it presents papules, burrows, nodules, or vesicles that can progress to excoriations and secondary infection, with persistent itch leading to sleep disturbance, psychological stress, and social stigma [3].

Pruritus in scabies arises from a multifaceted interplay of direct mite activity, host immune responses, and cutaneous barrier disruption [4]. One key contributor is the proteolytic enzymes excreted by *Sarcoptes scabiei*, which degrade keratin and facilitate mite burrowing into the stratum corneum [5]. Proteases can activate protease-activated receptors on keratinocytes and sensory neurons, leading to neurogenic inflammation and the amplification of itch signaling pathways [6]. Activation of these pathways with the release of pro-inflammatory cytokines such as IL-4, IL-13, and IL-31, perpetuates the pruritic cycle, further compromising the skin barrier and predisposing it to microbial colonization [7].

Emerging evidence suggests that *Sarcoptes scabiei* also modulates host microbial populations. An in vivo study using a porcine scabies model showed that scabies may alter the host skin microbiome and promote growth of opportunistic pathogens. Swe et al. showed an increase in *Staphylococcus* species on scabies-infected pigs, with a shift from the commensal *S. hominis* to the more virulent *S. chromogenes* [8,9]. Dysbiosis, in turn, may exacerbate inflammation and amplify pruritus through the release of microbial metabolites [5]. However, the precise interactions between scabies mites, their excretions, and the microbiome remain poorly understood, leaving a critical gap in our understanding of the pathophysiology of scabies lesions [10].

To address this knowledge gap, we performed shotgun metagenomic sequencing of skin samples collected from patients with scabies before and after treatment to characterize taxonomic composition, cross-domain microbial burden, and functional pathway potential [11,12]. This approach enabled comprehensive profiling of bacteria, fungi, viruses, and archaea within a single analytical framework. We aimed to characterize differences in microbial composition, anatomical site-associated community patterns, microbial burden, and functional potential between active scabies and the post-treatment state. The findings reported here provide a molecular foundation for future mechanistic and interventional studies investigating microbiome modulation in scabies management.

## 2. Results

### 2.1. Treatment-Associated Differences in Microbial Diversity and Community Structure

**Figure 1:**
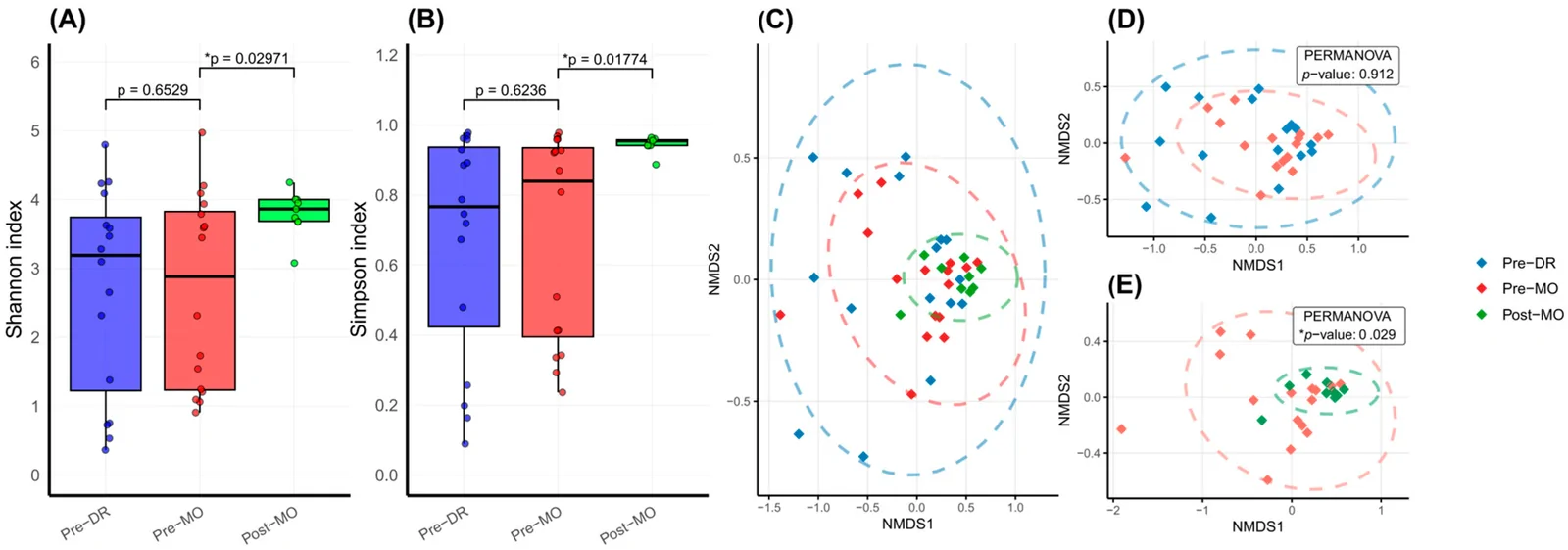
Alterations in skin microbial diversity associated with scabies infestation and treatment. (**A**,**B**) Pre-DR, pre-treatment dry-site samples; Pre-MO, pre-treatment moist-site samples; Post-MO, post-treatment moist-site samples. Comparison of microbial alpha diversity using Shannon and Simpson indices among Pre-DR (blue), Pre-MO (red), and Post-MO (green) groups. Statistical significance was determined by the Wilcoxon rank-sum test. (**C**) Non-metric multidimensional scaling (NMDS) plot based on Bray–Curtis dissimilarity, illustrating beta diversity across all groups. (**D**,**E**) Pairwise NMDS comparisons between Pre-DR and Pre-MO, and between Pre-MO and Post-MO. Significance is indicated as *p* < 0.05 (*).

Among pretreatment samples, alpha diversity and microbial community composition did not differ significantly between dry and moist sites (Shannon *p* = 0.6529; Simpson *p* = 0.6236; PERMANOVA *p* = 0.921). In exploratory group-level comparisons, Post-MO samples had higher alpha diversity than Pre-MO samples (Shannon *p* = 0.0297; Simpson *p* = 0.0177), and beta-diversity analysis showed a difference in community composition between these groups (PERMANOVA *p* = 0.029) (Figure 1A,B,E).

### 2.2. Bacterial and Viral Read-Based Burdens Were Lower in Post-Treatment Samples

**Figure 2:**
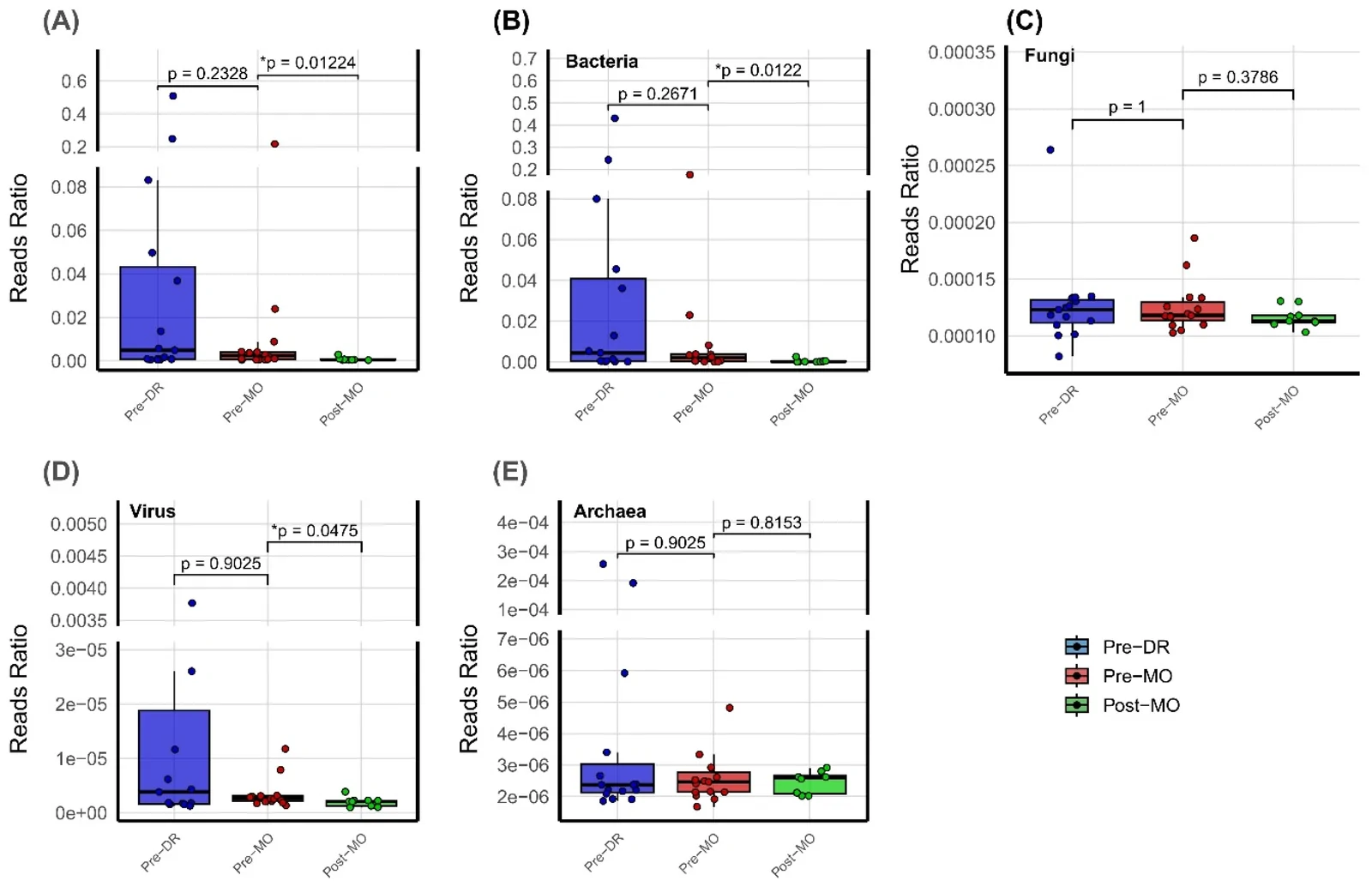
Changes in read-based microbial burden and domain-level proportions following scabies treatment. (**A**) Read-based microbial burden was calculated as the proportion of non-host and non-*Sarcoptes scabiei* reads among total sequencing reads across Pre-DR (blue), Pre-MO (red), and Post-MO (green) groups. (**B**–**E**) Relative abundances of bacterial, fungal, viral, and archaeal reads for each group. Asterisks indicate statistically significant differences (* *p* < 0.05) based on the Wilcoxon rank-sum test.

The read-based microbial burden, defined as the fraction of nonhost and non-*Sarcoptes scabiei* reads, was significantly lower in Post-MO than in Pre-MO (*p* = 0.0124). There was no significant difference between Pre-DR and Pre-MO (*p* = 0.228) (Figure 2A). Domain-level analysis showed that this reduction was primarily driven by decreases in bacterial and viral reads (Figure 2B,D). Both domains exhibited significant declines following treatment (*p* < 0.05 for bacteria and viruses), whereas fungal and archaeal abundances remained relatively unchanged (Figure 2C,E).

### 2.3. Differences in Dominant Bacterial and Fungal Taxa Before and After Treatment

**Figure 3:**
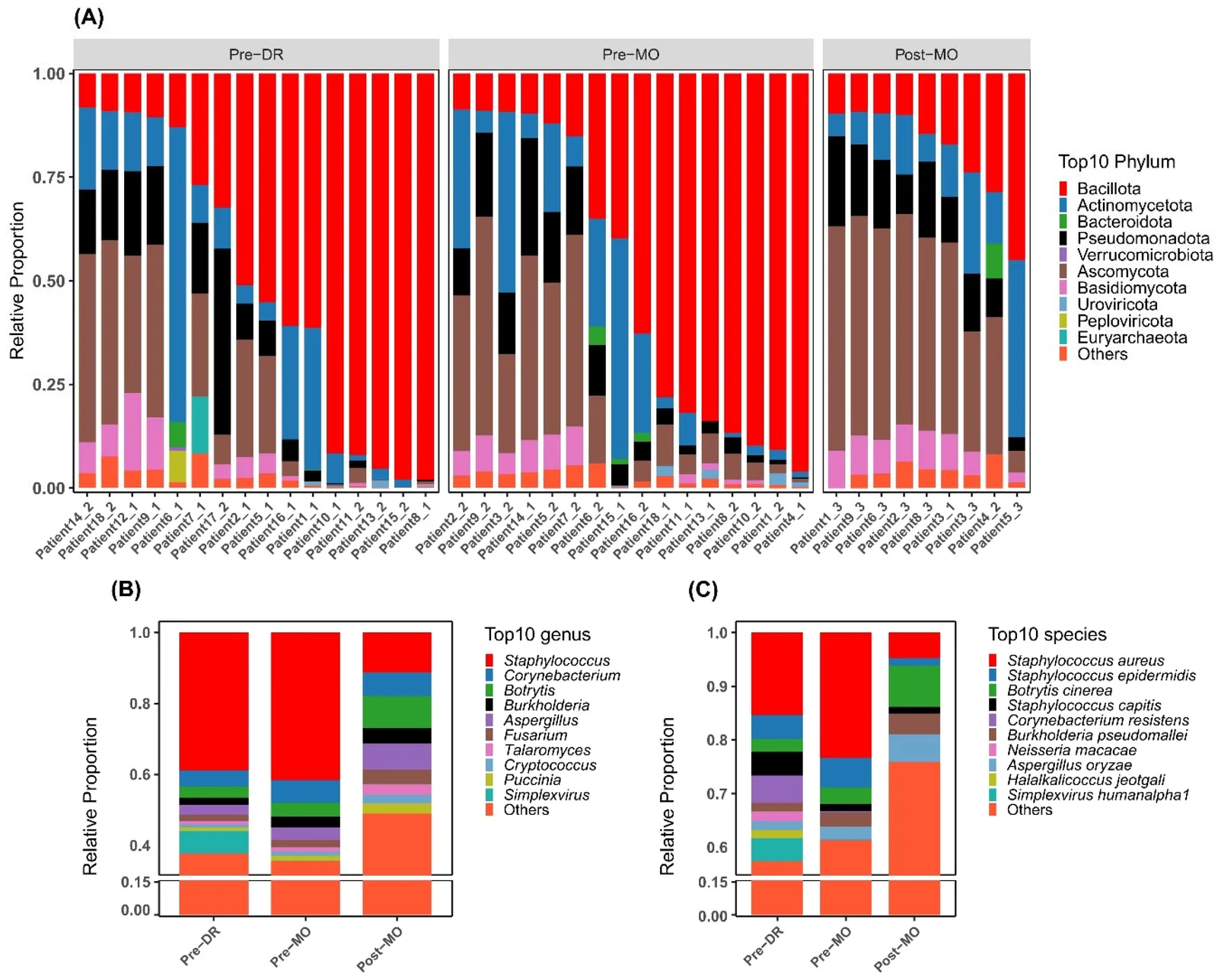
Taxonomic composition of the skin microbiome at multiple phylogenetic levels. (**A**) Stacked bar plots showing the relative abundance of the top 10 phyla across individual samples. (**B**) Average relative abundance of the top 10 genera for each clinical group. (**C**) Average relative abundance of the top 10 species for each clinical group. Data are presented for Pre-DR, Pre-MO, and Post-MO groups. Environment-associated assignments, including *Botrytis cinerea*, *Colletotrichum destructivum*, and *Lichenicola* species, are shown for transparency but should be interpreted cautiously because negative controls and dedicated contaminant-filtering procedures were not available.

**Figure 4:**
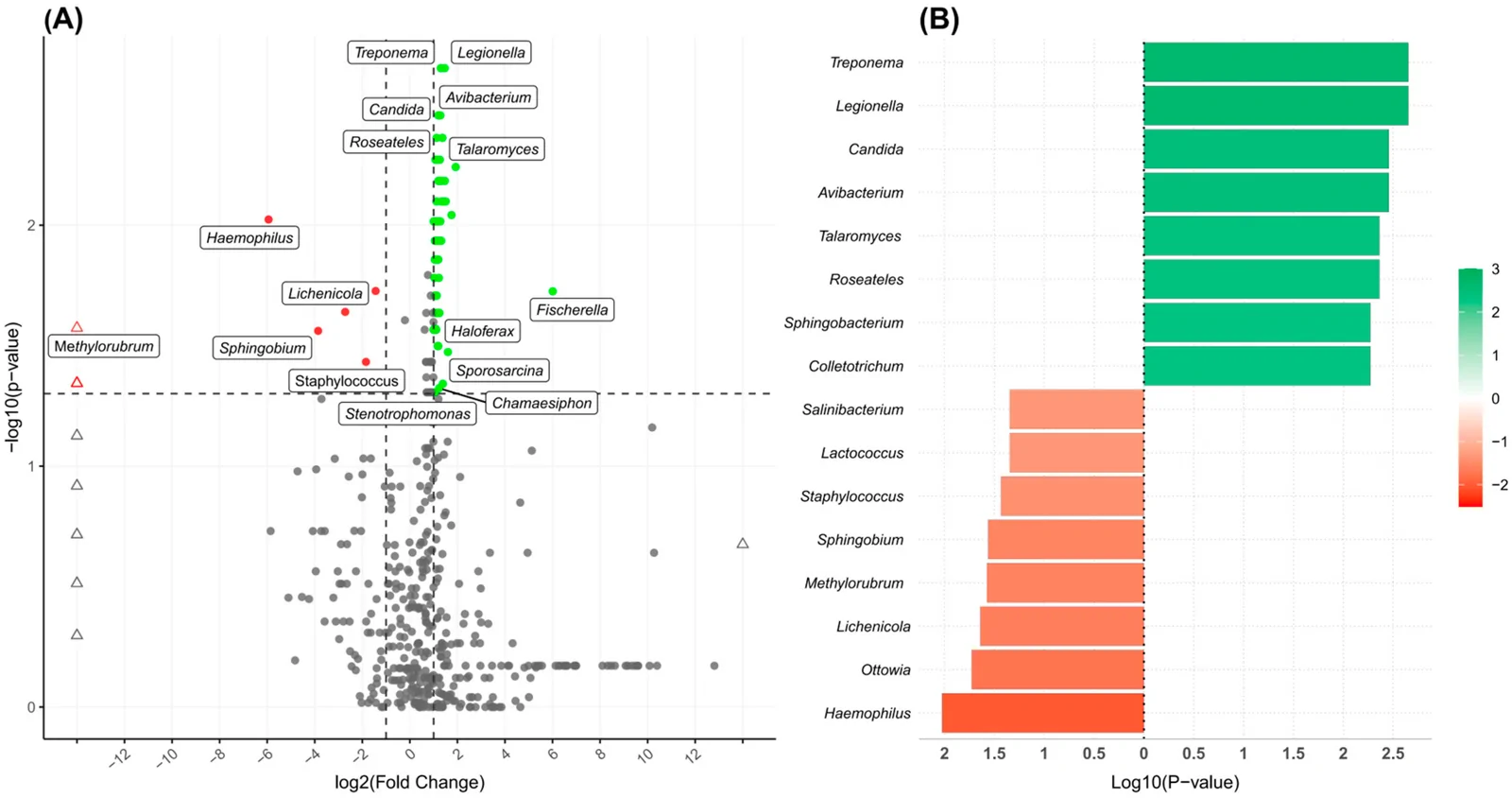
Exploratory genus-level differences based on unadjusted *p* values between Pre-MO and Post-MO samples. (**A**) Volcano plot showing genus-level differences between Pre-MO (negative log_2_ fold change, red) and Post-MO (positive log_2_ fold change, green). Selected genera are labeled; triangles at the plot margins indicate genera detected only in one group, for which finite log_2_ fold changes could not be calculated. (**B**) Eight genera per group are shown, ranked by −log_10_ of the unadjusted *p* value. Red and green bars indicate higher relative abundance in Pre-MO and Post-MO, respectively. Taxa were selected using nominal unadjusted *p* values for exploratory visualization. No genus-level taxon remained statistically significant after Benjamini–Hochberg FDR correction at a threshold of 0.05.

**Figure 5:**
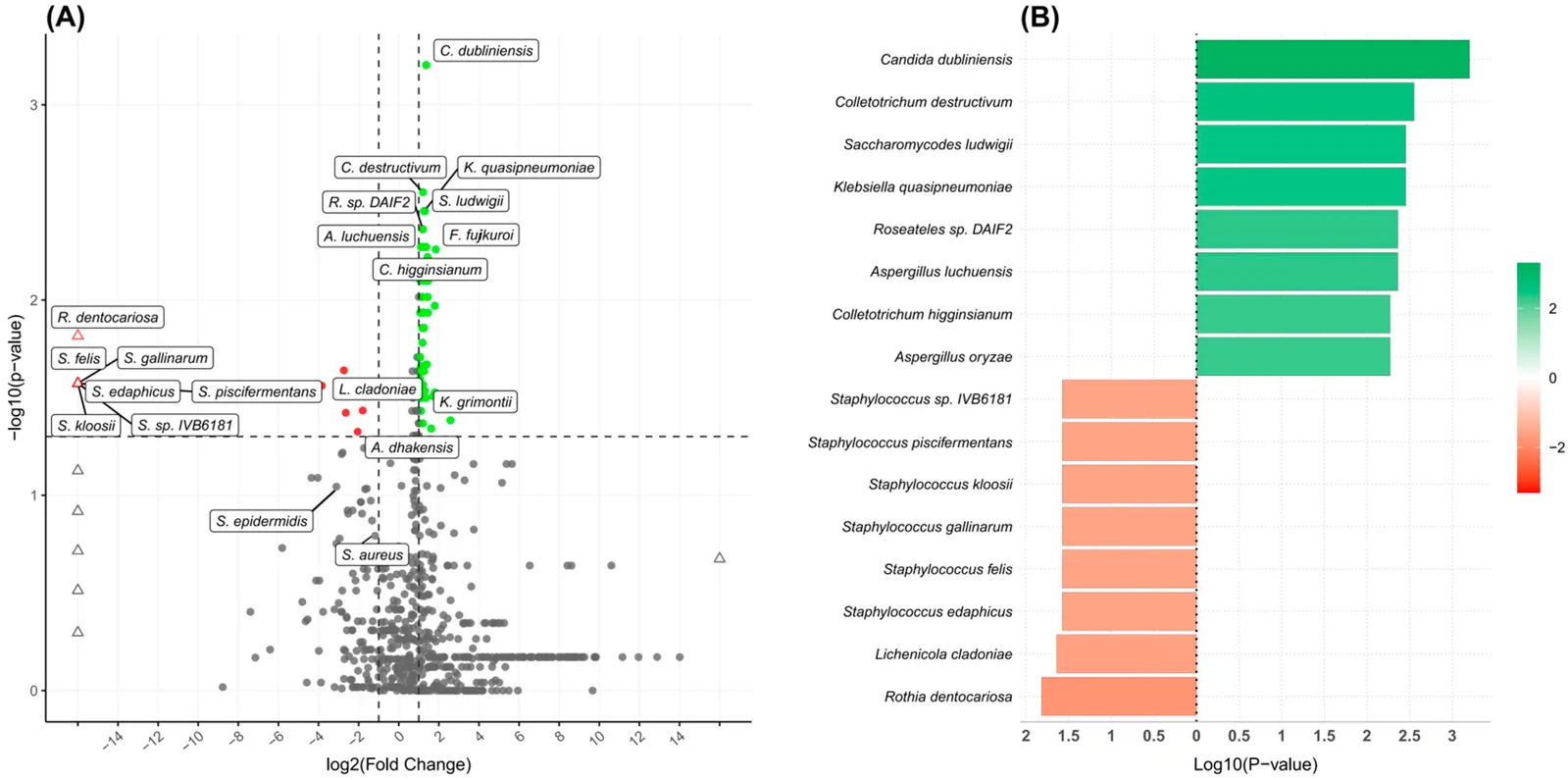
Exploratory species-level differences based on unadjusted *p* values between Pre-MO and Post-MO samples. (**A**) Volcano plot showing species-level differences between Pre-MO (negative log_2_ fold change, red) and Post-MO (positive log_2_ fold change, green). Selected species are labeled; triangles at the plot margins indicate species detected only in one group, for which finite log_2_ fold changes could not be calculated. (**B**) Eight species per group are shown, ranked by −log_10_ of the unadjusted *p* value. Red and green bars indicate higher relative abundance in Pre-MO and Post-MO, respectively. Taxa were selected using nominal unadjusted *p* values for exploratory visualization. No species-level taxon remained statistically significant after Benjamini–Hochberg FDR correction at a threshold of 0.05.

Taxonomic analysis of the three groups (Pre-DR, Pre-MO, and Post-MO) revealed differences in microbial community composition based on the top 10 taxa (Figure 3). At the phylum level, *Bacillota* was 31.94% more abundant in the pre-treatment groups compared with the post-treatment group, whereas *Ascomycota* was 22.04% more abundant in the post-treatment group compared with the pre-treatment groups. These descriptive patterns reflect differences in relative bacterial and fungal read proportions before and after treatment (Figure 3A). Despite these differences, *Bacillota*, *Actinomycetota*, and *Ascomycota* consistently dominated all groups. Before treatment, *Staphylococcus* was the dominant genus, and it remained the most abundant but decreased markedly after treatment (−33.58%). In contrast, reads assigned to *Botrytis* increased by 6.24% and represented the most abundant non-staphylococcal genus-level assignment in the post-treatment samples (Figure 3B). At the species level, *Staphylococcus aureus* was among the most abundant taxa, while a substantial proportion of reads was also assigned to the environment-associated species *Botrytis cinerea*. The latter assignment may represent environmental/background contamination or database-related misclassification. However, *S. aureus* (27.45%) and *S. epidermidis* (6.46%) were more abundant before treatment, whereas reads assigned to *B. cinerea* showed a 6.89% relative increase after treatment; however, because appropriate negative controls were unavailable, this finding was not interpreted as a confirmed biological change in the skin microbiome (Figure 3C). Exploratory differential abundance comparisons between the Pre-MO and Post-MO groups were based on unadjusted Wilcoxon rank-sum *p* values and the fold-change criteria described in Section 4.5. At the genus level, *Haemophilus*, *Ottowia*, and *Lichenicola* showed higher relative abundances in Pre-MO, while *Treponema*, *Legionella*, and *Candida* showed higher relative abundances in Post-MO at the nominal level (Figure 4A,B). At the species level, *Rothia dentocariosa*, *Staphylococcus edaphicus*, *Staphylococcus felis*, and *Staphylococcus gallinarum* showed nominal differences with higher relative abundances in Pre-MO and were largely undetected in Post-MO (Figure 5A). Conversely, *Candida dubliniensis*, *Colletotrichum destructivum*, and *Saccharomycodes ludwigii* showed nominal differences with higher relative abundances in Post-MO (Figure 5B). Among 1455 genera and 4111 species with valid *p* values, no taxon remained statistically significant after Benjamini–Hochberg FDR correction at a threshold of 0.05. The minimum adjusted *p* values were 0.594 at the genus level and 0.557 at the species level. No taxon met the FDR threshold when correction was repeated after retaining only taxa detected in at least two samples (Supplementary Table S2). The taxonomic patterns in Figure 4 and Figure 5 are therefore presented as exploratory observations.

### 2.4. Exploratory Differences in Predicted Nucleotide and Amino Acid Pathway Abundance

Of the 315 predicted pathways with valid *p* values, 99 had nominal unadjusted *p* < 0.05, but none remained statistically significant after BH-FDR correction (minimum adjusted *p* = 0.116). Selected nominal differences included higher relative abundances of adenosine deoxyribonucleotide de novo biosynthesis II, guanosine deoxyribonucleotide de novo biosynthesis II, and the superpathway of guanosine nucleotide de novo biosynthesis I in Pre-MO (unadjusted *p* = 0.0045 and adjusted *p* = 0.116 for each pathway). Other selected patterns involved L-valine biosynthesis, L-isoleucine biosynthesis I, and purine ribonucleoside degradation (Supplementary Figure S1). Restricting the correction to 201 pathways detected in at least two samples also yielded no FDR-significant pathway (minimum adjusted *p* = 0.074; Supplementary Table S2). These observations are exploratory and do not establish pathway-specific treatment-associated differences.

To describe the taxonomic contributions to these pathway profiles, we examined taxon-level contributions to functional abundance (Supplementary Figure S2). In the Pre-MO group, *Staphylococcus aureus*, *Corynebacterium accolens*, and *Staphylococcus epidermidis* consistently accounted for most pathway-associated reads across the selected predicted metabolic pathways. These descriptive contributions were examined for selected pathways and do not provide independent evidence of differential pathway abundance after FDR correction.

## 3. Discussion

This study identified a lower-diversity skin microbiome configuration during active scabies, characterized by no detected difference in diversity between pretreatment dry and moist anatomical sites, *Staphylococcus* predominance, and a higher nonhost, non-mite microbial read burden compared with post-treatment samples. Using shotgun metagenomic sequencing, we observed higher alpha diversity and a difference in community composition in post-treatment moist-site samples, together with a lower microbial read burden. These findings represent treatment-associated microbiome changes rather than evidence of restoration toward a healthy microbiome. Alpha diversity did not differ between Pre-DR and Pre-MO (Shannon *p* = 0.6529; Simpson *p* = 0.6236), and beta diversity likewise showed no separation (PERMANOVA *p* = 0.921). These results are compatible with reduced site-associated differentiation during active scabies in this cohort, although a nonsignificant comparison does not establish equivalence between anatomical sites. Post-treatment moist-site samples had higher alpha diversity and differed in community composition from pretreatment moist-site samples. Because post-treatment dry-site samples were excluded, recovery of dry–moist differentiation after treatment could not be evaluated. Similar site-overriding effects have also been reported in other inflammatory dermatoses, including psoriasis, where lesion status exerts a stronger influence than anatomical site [13].

From a read-based quantitative perspective, post-treatment samples had a significantly lower nonhost, non-mite microbial read burden than pretreatment samples, with the largest differences observed in bacterial and viral reads. Fungal and archaeal read fractions did not differ significantly between the pre- and post-treatment samples (*p* > 0.1). Nevertheless, several fungal taxa, including *Candida dubliniensis*, *Colletotrichum destructivum*, and *Saccharomycodes ludwigii*, showed higher relative abundances in Post-MO than in Pre-MO, a pattern that may reflect compositional effects associated with lower bacterial and viral read counts. Although permethrin has been reported to exert minimal or no direct antibacterial activity [14], the observed treatment-associated shifts may also be related to changes in skin barrier integrity or local immune activity following treatment. Because these host factors were not directly assessed, this mechanistic explanation remains hypothetical. Exploratory comparisons based on unadjusted *p* values suggested higher relative abundances of *Rothia dentocariosa*, *Staphylococcus edaphicus*, *Staphylococcus felis*, and *Staphylococcus gallinarum* before treatment. However, none of the genus- or species-level differences remained statistically significant after BH-FDR correction. *R. dentocariosa* and reads assigned to *Lichenicola cladoniae* showed large descriptive decreases, followed by multiple *Staphylococcus* species. *R. dentocariosa* is a commensal of the oral and respiratory tract with poorly defined associations with skin and no established link to dermatologic disease. Its relationship to skin colonization remains unclear. Only a few reports have described opportunistic infections caused by species of the *Rothia* genus [15]. In contrast, *L. cladoniae*, *Botrytis cinerea*, and *Colletotrichum destructivum* are primarily associated with environmental sources rather than established human skin microbiota. The use of standardized collection and processing procedures does not exclude contamination or batch effects, particularly in the absence of negative extraction and environmental controls. These observations may warrant further investigation but cannot distinguish biological variation from environmental background, reagent contamination, batch effects, or taxonomic misclassification.

In our study, *Staphylococcus* showed higher mean relative abundance before treatment at the nominal unadjusted *p* < 0.05 level, but this difference did not remain statistically significant after BH-FDR correction. Its descriptive predominance is consistent with a previous study of microbial changes following scabies infection in a porcine model [9]. This descriptive similarity warrants further investigation across host species. Both *S. aureus* and *S. epidermidis* showed lower relative abundances after treatment, but these species-level differences were not statistically significant. Both species are well-established contributors to eczematous inflammation and barrier disruption [16]. While *S. epidermidis* is generally commensal, it can acquire pathogenic traits under inflammatory conditions, including biofilm formation and protease secretion, that exacerbate barrier dysfunction [17]. The descriptive abundance patterns alone do not establish changes in microbial pathogenic activity after treatment.

Exploratory functional pathway profiling suggested a higher relative abundance of predicted purine biosynthesis pathways, particularly those involved in adenosine and guanosine nucleotide biosynthesis, in pretreatment samples. However, no predicted pathway remained statistically significant after BH-FDR correction, including in the analysis restricted to pathways detected in at least two samples. The pathway-specific interpretations are therefore hypothesis-generating. Taxonomically stratified profiles indicated consistent contributions from *Staphylococcus aureus*, *Staphylococcus epidermidis*, and *Corynebacterium accolens*. These findings represent differences in community-encoded functional potential and do not demonstrate increased purine metabolite production, transcriptional activity, or enzymatic activity. Alterations in skin metabolites, including those involved in purine metabolism, have been described in atopic dermatitis [18]. Experimental work in *S. aureus* has linked branched-chain amino acid transport to bacterial growth, membrane fatty acid composition, and virulence [19]. One possible interpretation is that changes in the skin environment during active scabies may be associated with microbial communities carrying greater purine biosynthetic potential. However, skin barrier function, keratin degradation, nutrient availability, and microbial metabolic activity were not directly measured in this study. *Corynebacterium accolens* has been reported to exhibit satellite growth dependent on lipid byproducts secreted by *S. aureus* [20]. In light of this observation, the pretreatment pathway profile may reflect differences in community composition and potential interspecies associations, including *Staphylococcus* predominance, rather than an effect attributable to a single taxon. The between-group differences in these predicted pathway profiles require confirmation in larger cohorts and do not establish functional normalization after treatment. Purine metabolism has been linked to virulence, persistence, and stress responses in *S. aureus* [21,22,23], whereas purinergic signaling has been implicated in regulating *Sarcoptes scabiei* behavior, including aggregation on host skin [24]. Although these previous findings raise the possibility of host–microbe–mite interactions involving purine-related processes, the present data do not establish a mechanistic link between microbial purine biosynthetic potential, purine metabolite availability, host responses, and mite behavior. This proposed linkage should therefore be regarded as a speculative hypothesis that requires validation using metabolomic, transcriptomic, or targeted functional studies.

Several methodological limitations should be considered when interpreting these findings. The cohort size was modest, and sampling was only partially paired; post-treatment dry-site samples were excluded owing to poor DNA quality. The exploratory group-level rank-sum comparisons did not account for intra-individual correlations among repeated samples. No genus- or species-level taxon or predicted pathway survived BH-FDR correction, limiting the evidence for individual treatment-associated microbial features. The absence of FDR-significant findings does not establish equivalence between groups. The reported log_2_ fold changes describe the magnitude and direction of relative-abundance differences, but confidence intervals were not reported, limiting assessment of their precision. Patients with documented systemic corticosteroid or cyclosporine use were retained in the analytic cohort. These concomitant treatments may have influenced the skin microbiome and represent potential confounders of the observed treatment-associated differences. The absence of healthy, age- and site-matched controls precluded determining whether the pretreatment microbial configuration differed from a healthy reference microbiome or whether the post-treatment configuration approached such a reference state. Accordingly, the observed pretreatment features cannot be considered specific to scabies and may also be associated with skin inflammation, participant age and clinical characteristics, or environmental exposures. Furthermore, the absence of an untreated temporal control group, together with a single post-treatment sampling point, limited our ability to distinguish treatment-associated microbiome shifts from natural temporal variation and to assess the stability or longer-term trajectory of the post-treatment microbial configuration. The observational pre–post design also does not establish whether the observed changes were attributable to permethrin treatment, resolution of infestation, or other concurrent factors. In addition, shotgun metagenomic sequencing characterizes community-encoded functional potential but does not directly measure gene expression, protein or enzymatic activity, metabolite abundance, or host responses. Low-abundance microbial domains, including viruses and archaea, may have been underrepresented because of sequencing-depth and reference-database limitations. Finally, the study was not designed or powered to directly assess associations between microbial features and clinical outcomes such as pruritus, lesion severity, or treatment response.

## 4. Materials and Methods

### 4.1. Specimen Collection and Subgroup Definition

Skin samples were collected from 18 Korean adult patients diagnosed and treated for scabies at the Department of Dermatology, Uijeongbu St. Mary’s Hospital, between September 2023 and December 2024. Scabies diagnosis was clinically established based on characteristic dermatological signs, such as burrows, erythematous papules, and excoriated nodules, and was confirmed by microscopic identification of *Sarcoptes scabiei* mites, eggs, or fecal pellets in skin scrapings.

Samples were categorized based on anatomical site of sample collection and treatment status. Dry regions included the hands and wrists, while moist regions included the axillary fossa, gluteal fold, and groin. Samples collected from dry regions after treatment (Post-DR) exhibited poor DNA quality with low DNA Integrity Number (DIN) values and were therefore excluded. The final analytic dataset included 41 samples in three groups: pre-treatment dry-site samples (Pre-DR, *n* = 16), pre-treatment moist-site samples (Pre-MO, *n* = 16), and post-treatment moist-site samples (Post-MO, *n* = 9). Among the moist-site samples used to assess treatment-associated changes, eight same-patient, same-site Pre-MO/Post-MO matched pairs were available, corresponding to 16 paired samples. These included five axillary fossa pairs and three gluteal fold pairs. One additional Post-MO axillary fossa sample did not have a corresponding pre-treatment sample from the same anatomical site and was considered unmatched. Because the dataset was only partially paired and imbalanced, treatment-associated changes were primarily assessed by comparing Pre-MO and Post-MO as exploratory group-level comparisons using all available samples. All patients were treated with 5% topical permethrin cream (Omeclean, Seoul, Republic of Korea) according to institutional practice. The treatment regimen consisted of three applications of permethrin. Post-treatment follow-up sampling was performed two weeks after completion of treatment, and all post-treatment samples were collected after microscopic confirmation of negative conversion for scabies mites, eggs, or fecal pellets. Concomitant medication use was reviewed retrospectively from medical records. No patients received systemic antibiotics or topical antibiotics during the relevant sampling period. Oral antihistamines, topical corticosteroids, emollients or barrier creams, systemic corticosteroids, and systemic immunosuppressants were recorded when documented and are summarized in Supplementary Table S1. Two patients received systemic corticosteroids during the relevant sampling period. Two patients had documented cyclosporine use. Patients with these concomitant treatments were retained in the analytic cohort. The mean age of the 18 participants was 72.1 ± 18.4 years, and female participants accounted for 72.2% (*n* = 13). There were no significant differences in age or sex distribution among the subgroups, and the patients exhibited diverse living conditions, ranging from walk-in outpatients to a majority of institutionalized individuals (Table 1). For microbiome analysis, specimens were collected by scraping the burrows and papules using sterile surgical blades until blood was visible, after confirming the presence of scabies mites via microscopic examination. Scrapings were then transferred onto sterile nylon-flocked swabs moistened with sterile saline. All collected samples were stored in sterile tubes and immediately placed in a refrigerator at 4 °C until DNA extraction. All sequencing reads were 151 base pairs (bp) in length [12].

### 4.2. Shotgun Sequencing Data Analysis

Shotgun metagenomic sequencing was performed to classify individual reads into their corresponding taxa, enabling the estimation of the relative abundance of microbial communities and the identification of associated metabolic pathways. Raw sequencing data for all samples were provided in FASTQ format. Initial quality control was conducted using Sickle (version 1.33), which removes low-quality reads by excluding sequences with a Phred quality score below 20 or a read length shorter than 100 base pairs [25]. This step ensured the retention of high-confidence reads by eliminating unreliable sequences from further analysis.

### 4.3. Taxonomic Classification and Diversity Analysis

Taxonomic classification was performed using Kraken2 (version 2.1.3) [26]. As whole-genome sequencing was conducted without an amplification step, a substantial proportion of host (*Homo sapiens*)-derived DNA sequences, along with reads originating from *Sarcoptes scabiei*, was anticipated. Each read was split into 35-base-pair k-mers and analyzed against a custom database built on the NCBI RefSeq framework, which included genomes from *Homo sapiens*, *Sarcoptes scabiei*, and a wide range of bacterial, fungal, viral, and archaeal taxa [27].

Following taxonomic classification with Kraken2, Bracken was applied to the Kraken2 classification output to improve the estimation of taxon abundance. Bracken re-estimated abundances at the designated taxonomic levels by redistributing reads assigned to higher taxonomic levels on the basis of database-derived k-mer distributions. Subsequent analyses were performed based on the microbiota, excluding *Homo sapiens* and *Sarcoptes scabiei*. Negative extraction controls, reagent blanks, and air or environmental controls were not included during sample processing, and no dedicated bioinformatic contaminant-removal procedure was applied. Consequently, environmental or reagent-derived contamination and database-related taxonomic misclassification cannot be excluded, particularly for environment-associated taxa. Such assignments were retained for transparency and interpreted cautiously.

Relative abundance data obtained from this taxonomic profiling were used to assess both alpha (α) and beta (β) diversity. Alpha diversity, representing microbial diversity within individual samples by accounting for both species richness and evenness, was quantified using the Shannon and Simpson indices [28]. Beta diversity, which evaluates differences in microbial community structure between groups, was calculated for the Pre-DR, Pre-MO, and Post-MO groups, and non-metric multidimensional scaling (NMDS) based on Bray–Curtis dissimilarity was applied to visualize dissimilarities in community composition among samples [29,30].

### 4.4. Metabolic Pathway Analysis

To investigate the metabolic functions of microbial communities, we utilized the HMP Unified Metabolic Analysis Network (HUMANN 3.0) [31]. HUMANN maps shotgun sequencing reads to known gene families and links them to metabolic pathways. Using taxonomic classification information, each read was assigned to specific pathways based on its amino acid sequence, referencing protein annotations from the UniRef database [32]. We used UniRef50 for functional annotation, which provides broader clustering than UniRef90 and compensates for limited pathway-level coverage by increasing the proportion of reads assigned to metabolic pathways [33]. The resulting pathway abundance profiles were used for comparisons between the Pre-MO and Post-MO groups.

### 4.5. Statistical Analysis

In this study, 41 skin microbiome samples collected from 18 scabies patients were categorized into three groups (Pre-DR, Pre-MO and Post-MO) based on anatomical site and treatment status. Because the dataset was only partially paired and imbalanced, primary analyses were conducted as exploratory group-level comparisons using all available samples. This approach preserved the available cohort structure but did not account for intra-individual correlations. Pairwise group comparisons of alpha diversity and read-based microbial burden were performed using the Wilcoxon rank-sum test [34]. All analyses were conducted using R software (version 4.3.3). Differences in overall microbial community composition were assessed by permutational multivariate analysis of variance (PERMANOVA) based on Bray–Curtis dissimilarity. PERMANOVA was performed using the adonis2 function in the vegan package with 999 permutations.

For the abundance comparisons presented in Figure 4A and Figure 5A, relative abundance data were used. For each taxon, the mean relative abundance was calculated separately for the Pre-MO and Post-MO groups. Log_2_ fold change was calculated as follows:

(1) $${log}_{2}\;FC\;=\;{log}_{2}\frac{\left(mean\;relative\;abundance\;in\;Post-MO\right)}{\left(mean\;relative\;abundance\;in\;Pre-MO\right)}$$

Accordingly, positive log_2_ fold-change values indicated higher abundance in Post-MO, whereas negative values indicated higher abundance in Pre-MO. The absolute magnitude of the log_2_ fold change was used to apply the fold-change cutoff, while its direction was retained to indicate the group with the higher mean relative abundance.

For taxonomic differential abundance analysis, no absolute-value transformation was applied to the log_2_ fold-change values, and no pseudocount was added during their calculation. Positive log_2_ fold-change values indicated higher abundance in Post-MO, whereas negative values indicated higher abundance in Pre-MO. Consequently, taxa detected exclusively in one group could not be assigned finite log_2_ fold-change values. These taxa were handled as a separate category and displayed as triangles at the corresponding margins of the volcano plots, with the direction determined by the group in which they were detected. (Figure 4A and Figure 5A).

Differential abundance comparisons between Pre-MO and Post-MO were performed using two-sided Wilcoxon rank-sum tests. *p* values were adjusted using the Benjamini–Hochberg false discovery rate (BH-FDR) procedure, with an adjusted *p* value < 0.05 considered statistically significant. For the full-set analysis, correction was applied separately across all valid *p* values within the genus-level (1455 tests), species-level (4111 tests), and functional pathway (315 tests) analyses. One pathway-table entry with a missing *p* value was excluded from correction. In an additional filtering analysis, BH-FDR correction was recalculated after retaining features detected in at least two samples, yielding 756 genera, 1564 species, and 201 pathways. The unadjusted *p* values of the retained features were unchanged. For exploratory visualization in Figure 4 and Figure 5, taxa were selected using nominal unadjusted *p* < 0.05 and at least a twofold difference in mean relative abundance between groups, corresponding to |log_2_ fold change| ≥ 1; taxa detected only in one group were handled separately as described above. Selected taxa were ranked by their unadjusted *p* values. These visualization criteria did not define significance after FDR correction. Selected pathway comparisons and taxonomically stratified contributions are presented as exploratory observations in Supplementary Figures S1 and S2. All statistical analyses were conducted using R software (version 4.3.3).

## 5. Conclusions

This study provides a shotgun metagenomic characterization of skin microbiome changes in patients with scabies before and after scabicidal treatment. Exploratory comparisons identified differences in microbial diversity, community composition, and read-based microbial burden. No genus- or species-level taxon or predicted pathway remained statistically significant after BH-FDR correction. Taxon- and pathway-specific observations should therefore be interpreted as exploratory. The findings do not establish restoration toward a healthy reference microbiome. Studies with matched controls, paired longitudinal sampling, and targeted functional validation are needed to determine their biological and clinical significance.

## Figures and Tables

**Table 1:** Clinical characteristics and sampling summary of the study cohort. Summary of 41 skin swab samples obtained from 18 adult scabies patients. Samples were collected from dry (hand, wrist) and moist (axillary fossa, gluteal fold) skin sites before and after standard scabicidal treatment. Pre-DR, Pre-MO, and Post-MO indicate pre-treatment dry-site, pre-treatment moist-site, and post-treatment moist-site samples, respectively. Detailed patient clinical information is provided in the condition column.

Sample	Gender/Age	Number	Treatment Status		Sampling Site	Condition
Patient 1	F/76	Patient1_1	Pre	Dry	Hand	Walk-in patient
		Patient1_2	Pre	Moist	Axillary fossa	
		Patient1_3	Post	Moist	Axillary fossa	
Patient 2	F/91	Patient2_1	Pre	Dry	Hand	Walk-in patient
		Patient2_2	Pre	Moist	Gluteal fold	
		Patient2_3	Post	Moist	Gluteal fold	
Patient 3	F/64	Patient3_1	Post	Moist	Axillary fossa	* Walk-in patient
		Patient3_2	Pre	Moist	Gluteal fold	
		Patient3_3	Post	Moist	Gluteal fold	
Patient 4	M/61	Patient4_1	Pre	Moist	Gluteal fold	Institutionalized patients
		Patient4_2	Post	Moist	Gluteal fold	
Patient 5	F/74	Patient5_1	Pre	Dry	Hand	Institutionalized patients
		Patient5_2	Pre	Moist	Axillary fossa	
		Patient5_3	Post	Moist	Axillary fossa	
Patient 6	F/70	Patient6_1	Pre	Dry	Hand	Institutionalized patients
		Patient6_2	Pre	Moist	Axillary fossa	
		Patient6_3	Post	Moist	Axillary fossa	
Patient 7	F/81	Patient7_1	Pre	Dry	Hand	Walk-in patient
		Patient7_2	Pre	Moist	Axillary fossa	
Patient 8	F/95	Patient8_1	Pre	Dry	Hand	Institutionalized patients
		Patient8_2	Pre	Moist	Axillary fossa	
		Patient8_3	Post	Moist	Axillary fossa	
Patient 9	F/89	Patient9_1	Pre	Dry	Hand	Institutionalized patients
		Patient9_2	Pre	Moist	Axillary fossa	
		Patient9_3	Post	Moist	Axillary fossa	
Patient 10	F/53	Patient10_1	Pre	Dry	Hand	Institutionalized patients
		Patient10_2	Pre	Moist	Axillary fossa	
Patient 11	F/81	Patient11_1	Pre	Moist	Gluteal fold	Institutionalized patients
		Patient11_2	Pre	Dry	Hand	
Patient 12	M/75	Patient12_1	Pre	Dry	Hand	* Walk-in patient
Patient 13	F/78	Patient13_1	Pre	Moist	Axillary fossa	Institutionalized patients
		Patient13_2	Pre	Dry	Hand	
Patient 14	M/22	Patient14_1	Pre	Moist	Axillary fossa	** Walk-in patient
		Patient14_2	Pre	Dry	Hand	
Patient 15	M/81	Patient15_1	Pre	Moist	Gluteal fold	Institutionalized patients
		Patient15_2	Pre	Dry	Hand	
Patient 16	F/94	Patient16_1	Pre	Moist	Gluteal fold	Institutionalized patients
		Patient16_2	Pre	Dry	Hand	
Patient 17	F/68	Patient17_1	Pre	Dry	Hand	Institutionalized patients
Patient 18	M/45	Patient18_1	Pre	Moist	Gluteal fold	Institutionalized patients
		Patient18_2	Pre	Dry	Hand	

* Affiliated with long-term care facility (self/family) ** Active-duty soldier.

## Data Availability

The shotgun metagenomic sequencing data generated in this study have been depositedin the National Center for Biotechnology Information (NCBI) Sequence Read Archive (SRA) under BioProject accession number PRJNA1521571. The BioProject record is available at https://www.ncbi.nlm.nih.gov/sra/PRJNA1521571 (accessed on 14 September 2026).

## Supplementary Materials

The following supporting information can be downloaded at https://www.mdpi.com/article/10.3390/ijms27188397/s1.
