# Supplementary material for: Shotgun Metagenomic Characterization of Skin Microbiome Shifts in Human Scabies Before and After Scabicidal Treatment
Source: Int J Mol Sci. 2026 Sep 21;27(18):8397. doi: 10.3390/ijms27188397 (PMC13607506; doi:10.3390/ijms27188397)
Supplement: Supplementary file 1 [file ijms-27-08397-s001.zip › ijms-4463435-supplementary.pdf]

Supplementary Materials

Supplementary Table S1. Concomitant medications and supportive treatments documented during the relevant sampling period.

Patient	Topical corticosteroid	Systemic corticosteroid	Oral antihistamine or antipruritic	Emollient or barrier cream	Systemic immunosuppressant
Patient 1	Desowen	No	Bepotastine, Azelastine	No	No
Patient 2	Topisol	No	Levocetirizine, Hydroxyzine	Yes	No
Patient 3	Topisol	No	Levocetirizine, Hydroxyzine	Yes	No
Patient 4	Topisol	No	Levocetirizine, Hydroxyzine	Yes	No
Patient 5	Desowen	No	Bepotastine, Azelastine	No	No
Patient 6	Desowen	No	Bepotastine, Azelastine	No	No
Patient 7	Topisol	No	Levocetirizine, Hydroxyzine	Yes	No
Patient 8	Desowen	No	Bepotastine, Azelastine	No	No
Patient 9	Topisol	No	Levocetirizine, Hydroxyzine	Yes	No
Patient 10	Desowen	No	Bepotastine, Azelastine	No	No
Patient 11	No	Triamcinolone	Bepotastine, Levocetirizine	No	No
Patient 12	No	No	Bepotastine, Azelastine	No	No
Patient 13	Topisol	No	Levocetirizine, Hydroxyzine	Yes	No
Patient 14	Topisol	Prednisolone	Levocetirizine, Bepotastine	No	No
Patient 15	Desowen	No	Levocetirizine, Hydroxyzine	Yes	No
Patient 16	No	No	Levocetirizine, Hydroxyzine	No	Cyclosporine
Patient 17	Topisol	No	Levocetirizine, Hydroxyzine	Yes	No
Patient 18	Topisol	No	Levocetirizine, Hydroxyzine	No	Cyclosporine

Note: Systemic antibiotics and topical antibiotics were not documented in any patient during the relevant sampling period.

Supplementary Table S2. Summary of multiple-testing correction for genus, species, and predicted pathway comparisons.

Analysis	Feature set	Valid tests	Unadjusted P < 0.05	Minimum adjusted P	Adjusted P < 0.05
Genus	Full set	1,455	85	0.5940	0
Genus	Detected in ≥2 samples	756	85	0.3292	0
Species	Full set	4,111	104	0.5575	0
Species	Detected in ≥2 samples	1,564	104	0.4440	0
Pathway	Full set	315	99	0.1164	0
Pathway	Detected in ≥2 samples	201	99	0.0742	0

BH-FDR correction was applied separately to each analysis and feature set. The full pathway table contained 316 entries, of which 315 had valid P values; one entry with a missing P value was excluded. The additional filtered analyses retained features detected in at least two samples and recalculated BH-adjusted P values across the retained tests. The original unadjusted P values were unchanged. Counts of nominal findings in this table use P < 0.05 alone and do not apply a fold-change criterion. No analysis yielded an adjusted P value < 0.05.

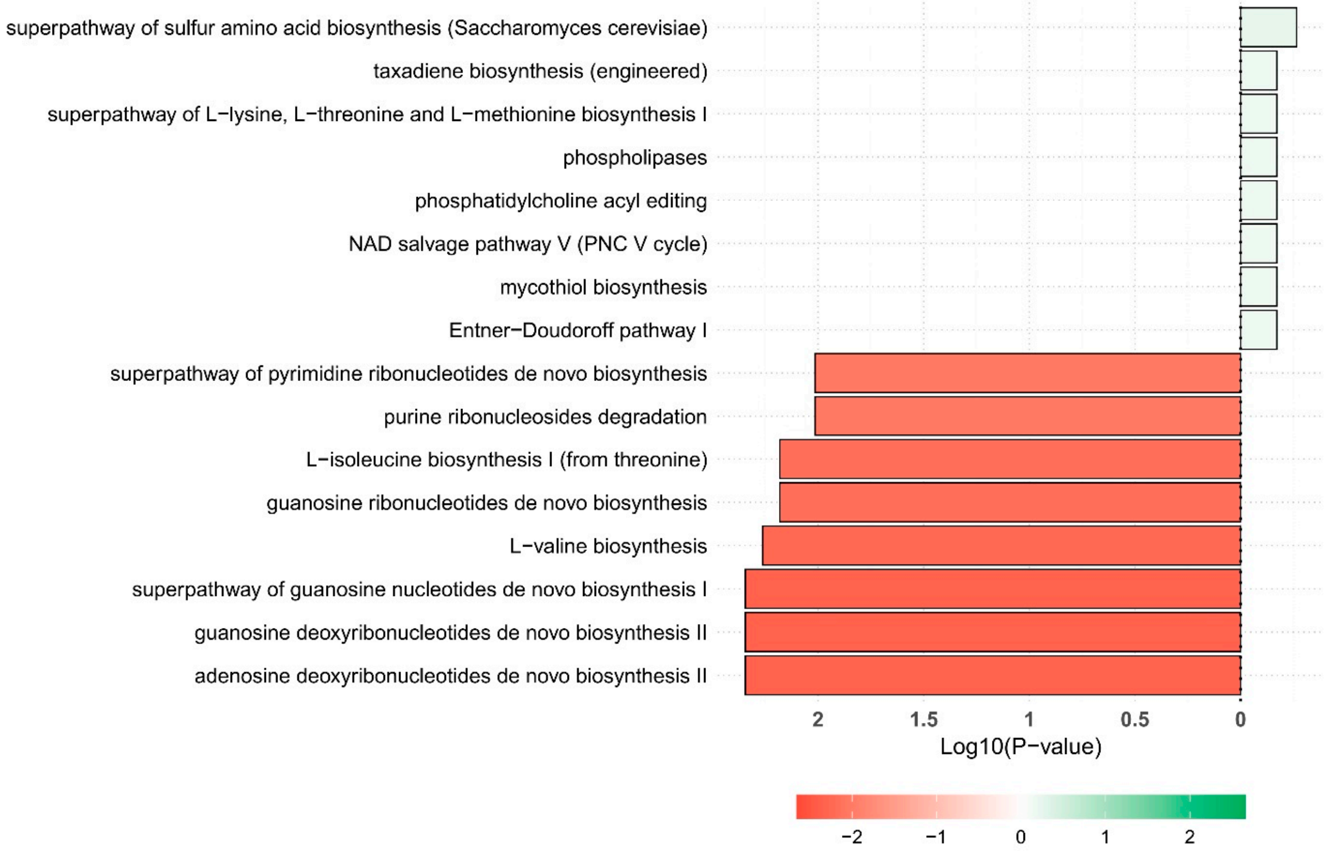

Supplementary Figure S1. Exploratory differences in predicted microbial pathway abundance between Pre-MO and Post-MO. Selected pathway comparisons are displayed using unadjusted Wilcoxon rank-sum P values. Absolute bar length corresponds to $-\log_{10}(P)$, with red bars extending left for higher abundance in Pre-MO and green bars extending right for higher abundance in Post-MO. The displayed pathways include comparisons that do not meet nominal P < 0.05. None of the 315 pathways with valid P values remained statistically significant after BH-FDR correction (minimum adjusted P = 0.116); none met the 0.05 threshold in the analysis restricted to 201 pathways detected in at least two samples (minimum adjusted P = 0.074). The figure is provided for exploratory description only.

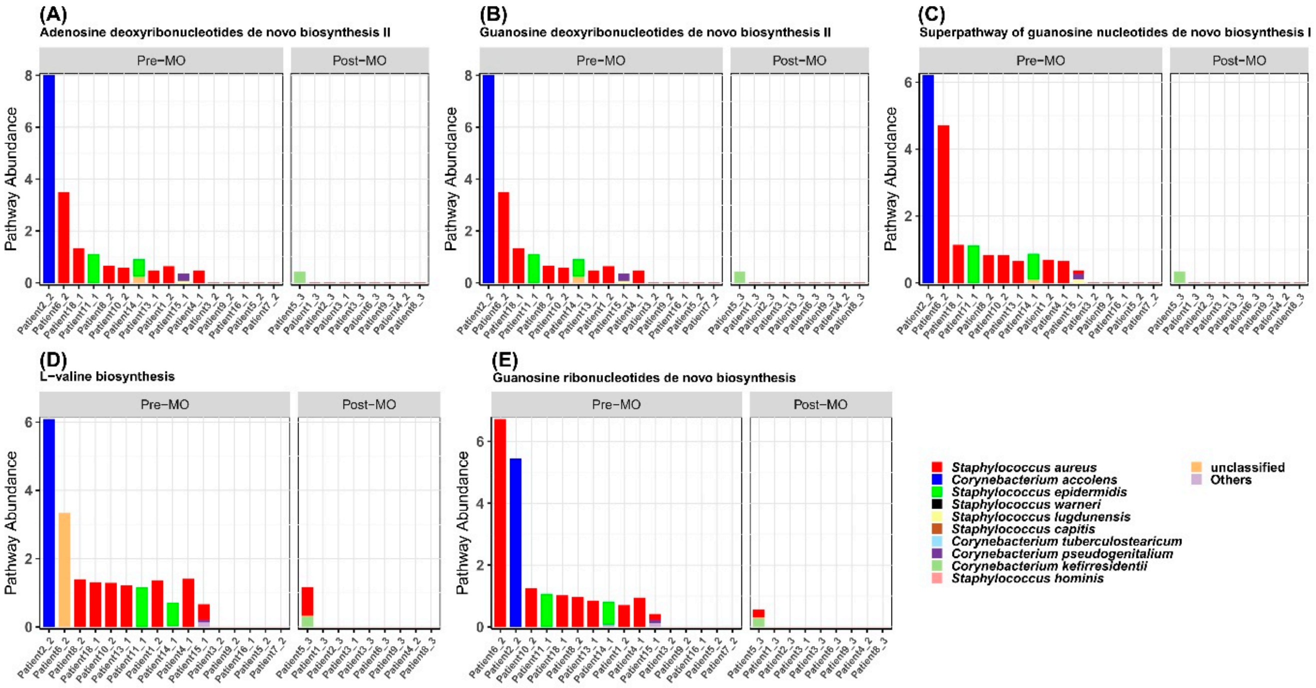

Supplementary Figure S2. Taxon-level contributions to selected predicted metabolic pathways. Stacked bars show pathway abundance contributions for individual Pre-MO and Post-MO samples. Panels show (A) adenosine deoxyribonucleotides de novo biosynthesis II, (B) guanosine deoxyribonucleotides de novo biosynthesis II, (C) the superpathway of guanosine nucleotides de novo biosynthesis I, (D) L-valine biosynthesis, and (E) guanosine ribonucleotides de novo biosynthesis. Colors distinguish the ten displayed contributing taxa, unclassified contributions, and other taxa. These pathways were selected for exploratory characterization, and none remained statistically significant after BH-FDR correction. The taxonomic contributions are descriptive and do not establish pathway activation or a treatment effect.
